# Stimuli-responsive nanoplatforms for precision activation of the STING pathway in cancer immunotherapy

**DOI:** 10.3389/fimmu.2026.1714249

**Published:** 2026-01-26

**Authors:** Dongming Bi, Xue Yang, Yuan Wang, Jiangyan Yong, Fujie Yang, Xiaoyu Tan, Yuchen Li, Dongming Zheng, Pandeng Li

**Affiliations:** 1 Department of Laboratory Medicine, Hospital of Chengdu University of Traditional Chinese Medicine, Chengdu Sichuan, China; 2 Department of Laboratory Medicine, Deyang Hospital Affiliated Hospital of Chengdu University of Traditional Chinese Medicine, Deyang Sichuan, China; 3 Nuclear Medicine Department, Hospital of Chengdu University of Traditional Chinese Medicine, Chengdu Sichuan, China; 4 Department of Nuclear Medicine, Yaan People’s Hospital, Ya an, China

**Keywords:** antitumor immunity, cancer immunotherapy, cGAS-STING signalling, drug delivery, immune activation, stimuli-responsive nanoplatforms, STING agonist, tumour microenvironment

## Abstract

The stimulator of interferon genes (STING) pathway plays a unique role in antitumor immunity, bridging innate and adaptive immune responses to initiate a sustained and highly effective antitumor immune response. However, due to the widespread expression of the STING pathway and the lack of clearly distinguishable physiological and pathological features, its excessive or systemic activation can trigger severe adverse effects, such as cytokine storms, thereby limiting its clinical applicability. With the development of nanotechnology, stimuli-responsive nanoplatforms designed based on tumor microenvironment (TME) signals (such as pH, glutathione, reactive oxygen species, hypoxia, and enzymes) and exogenous stimuli (including light, ultrasound, radiation, and magnetic fields) provide a promising strategy for the precise activation of the STING pathway. These nanoplatforms can achieve tumor-specific and controllable STING activation, thereby minimizing off-target toxicity, and can be combined with chemotherapy, radiotherapy, or photodynamic therapy to produce multimodal synergistic antitumor effects. Here, we provide a systematic overview of stimuli-responsive nanoplatforms for STING activation, highlighting their design strategies and how they can reverse immunosuppressive TME through STING pathway activation. Additionally, we discuss the challenges facing their clinical translation and outline future directions, aiming to provide a foundation for further research in this field. In conclusion, stimuli-responsive STING-activating nanoplatforms demonstrate significant potential in antitumor therapy and may serve as a novel therapeutic strategy.

## Graphical Abstract

**Figure:**
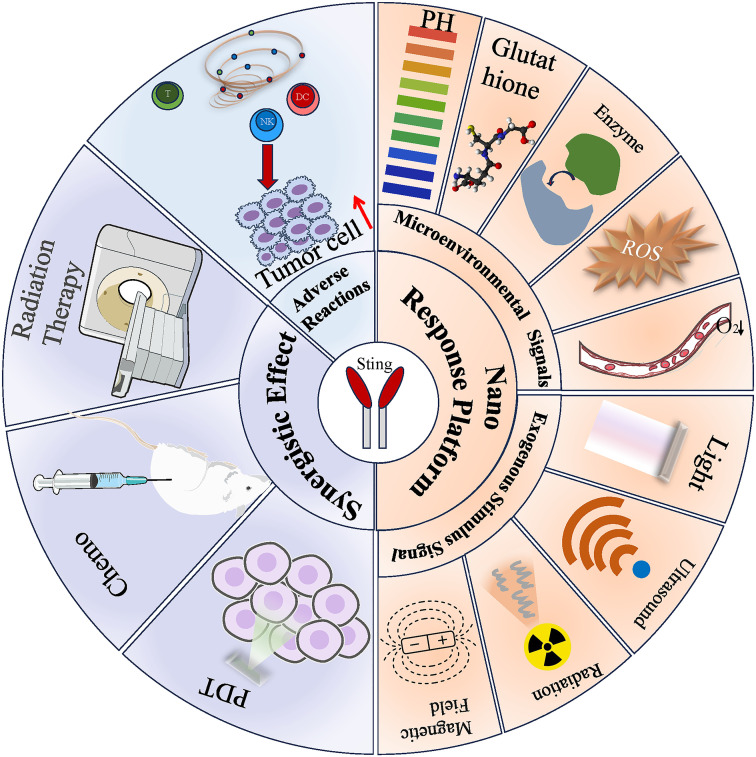

## Highlights

1. Stimuli-responsive nanoplatforms can respond to endogenous signals and exogenous stimuli, thereby enabling precise, tumour-specific, controllable, and low-toxicity activation of the STING pathway.
2. Stimuli-responsive nanoplatforms can both alleviate the immunosuppressive tumour microenvironment by depleting glutathione, reactive oxygen species, and so on, and synergise with other therapies such as photodynamic therapy to enhance antitumor efficacy.
3. Future studies should focus on optimising material design, conducting systematic safety assessments, integrating combination therapy strategies, and performing *in vivo* experiments to evaluate long-term safety and clinical efficacy, thereby guiding translational applications.

## 1 Introduction

The silencing or activation of the immune system is a pivotal determinant in tumour initiation, progression, and metastasis, profoundly influencing conventional cancer therapies. For instance, radiotherapy can induce the release of tumour-associated antigens, promote the maturation of antigen-presenting cells (APCs) and activate effector T cells, thereby eliciting an “abscopal effect,” in which untreated lesions also exhibit clinical responses (1). However, driven by myeloid-derived suppressor cells, tumour-associated macrophages, and regulatory T cells, along with adverse factors such as hypoxia and acidity, the tumour microenvironment (TME) develops a markedly immunosuppressive profile (2). Therefore, converting the TME from “cold” to “hot” has become a key strategy in cancer therapy, capable of directly eliciting antitumor effects while markedly enhancing the efficacy of conventional treatments. In recent years, cancer immunotherapies, including immune checkpoint inhibitors (ICIs), bispecific T-cell engagers, and chimeric antigen receptor T cells, have achieved significant clinical breakthroughs in treating solid tumours. However, overall patient survival benefits remain limited, with response rates typically ranging from 10% to 30% (3, 4). Indeed, tumour-specific T cell activity critically depends on intercellular signalling and regulation by innate immunity, which mediates tumour immune surveillance through the recognition, suppression, and clearance of tumour cells. Therefore, effective bridging and coordination between innate and adaptive immunity are essential for achieving durable tumour control (5).

**Figure 1:**
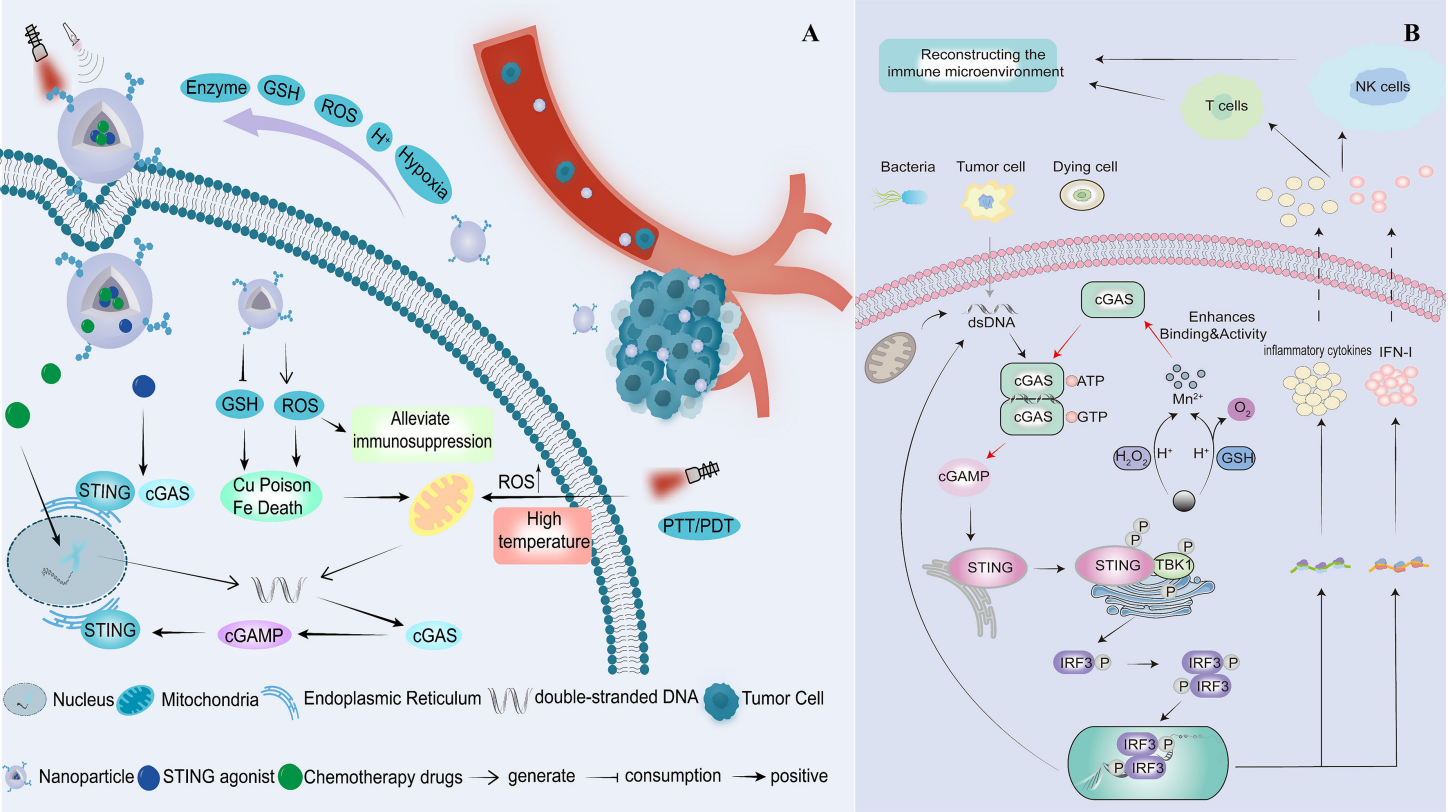
**(A)***In vivo* release mechanism of the stimuli-responsive nanoplatform. **(B)** Reprogramming the immunosuppressive microenvironment through activation of the STING pathway.

The cyclic GMP–AMP synthase (cGAS)–stimulator of interferon genes (STING) pathway is a crucial component of the innate immune system. By recognising aberrant intracellular DNA, it activates innate immunity and bridges to adaptive immunity, thereby enhancing antigen immunogenicity (6). cGAS binds double-stranded DNA (dsDNA) in a sequence-independent manner, activating itself to produce the second messenger cyclic GMP–AMP (cGAMP). cGAMP then activates STING, triggering its translocation from the endoplasmic reticulum to the Golgi apparatus and recruiting TANK-binding kinase 1 (TBK1). TBK1 phosphorylates the transcription factor interferon regulatory factor 3, which drives its nuclear translocation and induces the production of type I interferons and inflammatory cytokines. These signals regulate the release and presentation of antigens, enhance the cytotoxic activity of natural killer cells, modulate T-cell responses, and collectively reshape the immune microenvironment to eliminate tumours (5, 7). Numerous studies have shown that activation of the STING pathway can induce immune-mediated tumour cell death and improve the control of primary and metastatic tumours by promoting the maturation of dendritic cells (DCs) and antigen-specific CD8^+^ T-cell responses (8). Furthermore, combining STING agonists with radiotherapy, ICIs, or chimeric antigen receptor T-cell therapy has been shown to produce pronounced synergistic effects. Compared with any monotherapy, these combination treatments not only enhance tumour control but also reverse tumour resistance to conventional therapies (9). However, these STING agonists currently face significant limitations before clinical application, including low bioavailability, limited membrane permeability, suboptimal tumour accumulation, and the risk of dose-dependent cytokine storms upon systemic administration (10). To address these challenges, stimuli-responsive nanoplatforms have shown great promise. These nanocarriers can respond to TME cues, such as acidity, reactive oxygen species (ROS), enzymatic activity, and glutathione (GSH), or to external stimuli, such as light, heat, and ultrasound, enabling tumour-specific drug release while effectively reducing systemic toxicity (11, 12). Moreover, nanocarriers can directly induce cytotoxic effects through therapies such as photothermal or photodynamic approaches, thereby synergistically activating the immune system and facilitating the clearance of tumour cells (12). Against this backdrop, we systematically review the latest advances in stimuli-responsive nanodelivery systems designed to activate the STING pathway, focusing on their design strategies and mechanisms of action (**Figure 1**), and providing an outlook on current challenges and future development directions.

## 2 Clinical translation research progress of STING agonists

Over the past decade, as understanding of the STING pathway’s pivotal role in cancer immunotherapy has deepened, STING agonists have gradually emerged as a promising alternative to conventional cancer treatments. STING agonists currently in clinical research primarily fall into the following categories: synthetic cyclic dinucleotides (CDN), non-CDN small molecules, antibody-drug conjugates, indirect STING agonists (13). CDN was the first STING agonist to enter drug development. However, its highly hydrophilic backbone and negative charge resulted in poor bioavailability, limited cellular uptake, and a propensity for adverse events, ultimately demonstrating limited therapeutic benefit in clinical trials (14). For example, a Phase I clinical trial evaluated the efficacy of IMSA101 as monotherapy and in combination with an ICIs. While some degree of tumour shrinkage was observed across all treatment groups, no responses were observed in the monotherapy group according to RECIST criteria, and only one partial response was achieved in the combination group. Additionally, in the combination therapy group, 11 subjects reported a total of 23 treatment-related adverse events, including one case of cytokine release syndrome (15). Furthermore, in two Phase I clinical trials evaluating ADU-S100 as a monotherapy and in combination treatment strategies, the drug demonstrated only minimal clinical benefit, a stark contrast to the significant efficacy observed in its preclinical studies. Studies indicate that ADU-S100 has no discernible effect on macrophage recruitment, with tumour growth inhibition and systemic immune activation observed only in a small number of patients (16, 17). Nevertheless, multiple CDN derivatives—including BMS-986301, BI 1387446, MK-1454, and SB 11285—have demonstrated encouraging results in preclinical studies. These compounds have now advanced to Phase I clinical trials to evaluate their safety, tolerability, and antitumor activity, with the expectation of achieving substantial breakthroughs in clinical practice. Interested readers may refer to recent reviews by Nerdinger et al., Hines et al., and Wen et al., which comprehensively outline the clinical development progress of STING agonists (9, 18, 19).

To overcome the limitations of CDN-type STING agonists, researchers have begun developing non-CDN small-molecule agonists with higher serum stability, improved tissue penetration, and the potential for systemic administration (20). DMXAA was the first such drug to enter Phase III clinical trials, but its development was discontinued due to insufficient efficacy. In Phase III clinical trials for non-small cell lung cancer, the combination therapy of DMXAA with paclitaxel/carboplatin demonstrated no significant advantage over paclitaxel/carboplatin monotherapy in terms of objective response rate, progression-free survival, or overall survival (21). The fundamental reason lies in the drug’s high affinity for the STING receptor in mice, coupled with its limited capacity to effectively activate all five STING subtypes in humans (9, 22). Nevertheless, these findings have spurred the ongoing development of non-CDN STING agonists. Small molecules, such as ABZI-class compounds, MSA-2, SR-717, MK-2118, TAK-676, and E7766, have emerged, demonstrating significant efficacy in preclinical studies through favourable cellular uptake, tumour retention, and effective activation of human STING (18, 22). For example, MSA-2 was designed as a prodrug to activate STING. Under the acidic conditions of the TME, MSA-2 transitions from a monomeric form to a non-covalent dimerised form, powerfully activating STING, whereas the monomeric form exhibits no activity. Although MSA-2 enhances natural killer cell killing activity and promotes T cell infiltration in mice, monotherapy only delays tumour growth without achieving complete tumour clearance (23). However, MSA-2 demonstrates superior efficacy in combination therapy. For example, MSA-2, combined with platinum-based drugs, more effectively shrinks tumours and prolongs survival in the B16F10 model compared to either agent alone (24). Ji et al. also found that combining MSA-2 with the anti-TGF-β/PD-L1 bispecific antibody YM101 significantly elevated proinflammatory and chemokine levels in the TME. This enhanced antigen presentation and promoted lymphocyte infiltration, leading to more effective suppression of tumor growth (25). However, non-CDN also failed to demonstrate the anticipated pharmacodynamic or clinical efficacy in Phase I clinical trials. Luke et al. evaluated the effectiveness of MK-2118 in combination with pembrolizumab in patients with advanced solid tumours or lymphoma. The study demonstrated that while both intratumoral and subcutaneous administration exhibited manageable toxicity, antitumor activity was limited. More importantly, only intratumoral administration induced systemic immune effects (26). The Phase I clinical trial of E7766 also demonstrated good tolerability with intratumoral administration, with some patients achieving disease stabilisation. However, its pharmacodynamic effects showed no dose dependency, and the increase in immune factors was transient (27). Therefore, non-CDN drugs still face numerous challenges, including whether they can effectively penetrate the TME after systemic administration, and the relationship between dosage, toxicity, and potential benefits remains unclear.

In addition to the two aforementioned classes of agonists, metal ions (such as Mn, Zn, and Co), dsDNA, and bacterial vectors can also activate STING. Metal ions not only promote ROS production through Fenton-like reactions, leading to DNA damage and apoptosis in tumour cells, but also directly bind to cGAS, inducing a conformational change in this protein. This enhances cGAS’s recognition and stable binding to cytoplasmic dsDNA, thereby promoting cGAMP generation (28, 29). In preclinical studies, these metal ions have demonstrated significant antitumor potential (30). For example, combining Mn with YM101 can reshape tumours from immune-excluded/suppressed states to inflammatory states, effectively controlling tumour growth and prolonging survival in tumour-bearing mice (31). However, no metal ion-based STING agonists have yet entered clinical trials. More importantly, metal ions and the aforementioned STING agonists exhibit non-specific distribution within the body, lack tumour-targeting capabilities, and have dose-limiting toxicity issues. These problems may induce cytokine storms or cause liver and kidney damage, severely limiting their clinical application (32). Therefore, the current research focus is gradually shifting toward enhancing the tumour selectivity, persistence, and safety of STING agonists. Nanodelivery systems based on receptor expression differences, TME characteristics, or responses to exogenous stimuli have emerged as a promising strategy. The bacterial vector STING agonist SYNB1891 is an *Escherichia coli* Nissle strain genetically engineered to express cAMP in hypoxic TMEs and localise within them. In Phase I clinical trials, SYNB1891 demonstrated significant activation of the STING pathway but resulted in four cases (4/28) of cytokine release syndrome events (33). Research on exoSTING further demonstrated the efficacy of nanocarriers. ExoSTING is an engineered exosome that highly expresses the glycoprotein PTGFRN on its surface, enabling it to specifically target APCs and be internalized by them. Researchers found that CDN-loaded exoSTING effectively retained at tumor injection sites, strongly activating the STING pathway without systemic exposure, thereby inducing pro-inflammatory changes in the TME (34). Consequently, more precise, controllable, and safe nanodelivery strategies represent a key direction for overcoming clinical bottlenecks in STING agonists.

## 3 pH-responsive nanoplatforms

The TME comprises stromal cells, immune cells, fibroblasts, growth factors, blood vessels, and extracellular matrix components, collectively forming the primary structural and functional foundation of solid tumors alongside tumor cells (35). Due to the elevated energy and oxygen demands of rapidly proliferating tumour cells, the TME often experiences hypoxia, which is further exacerbated by the structural abnormalities and inadequate perfusion of the newly formed vasculature. Under these circumstances, tumour cells predominantly rely on glycolysis to meet their energy needs (Warburg effect), leading to lactate accumulation and the formation of an acidic microenvironment (2). This intrinsic acidity provides a natural trigger for pH-responsive nanoplatforms, making pH-responsive design one of the most widely employed strategies to enable site-specific and controlled drug delivery (36). This strategy achieves targeted drug release by introducing pH-sensitive chemical bonds and non-covalent interactions, or by inducing acid-triggered physicochemical transformations of the carrier (e.g., dissolution, structural collapse, or surface charge conversion).

### 3.1 pH-responsive carrier-free nanoplatform

pH-responsive carrier-free nanoplatforms are typically constructed through non-covalent interactions (such as electrostatic interactions, hydrogen bonds, and van der Waals forces) and acid-sensitive labile bonds (such as coordination bonds) (36). These interactions enable nanoparticles to remain stable under physiological conditions while undergoing controlled disassembly within the acidic TME, thereby achieving precise release of therapeutic drugs. Raddeanin A (RA) is a natural compound capable of activating the STING pathway by inducing mitochondrial DNA (mtDNA) leakage. RA forms nanocomposites (FANPs) with FeCl_3_ through the formation of RA–Fe^3+^ coordination bonds. FANPs remain highly stable in the bloodstream but rapidly depolymerise under the acidic conditions of the TME. The released Fe^3+^ can repolarise pro-tumorigenic M2 macrophages into anti-tumour M1 macrophages, while RA further enhances the M1 phenotype by activating the STING pathway, thereby synergistically inducing a potent anti-tumour immune response. In an immunosuppressive 4T1 breast cancer model, FANPs significantly inhibited tumour growth. They also demonstrated strong STING activation in patient-derived tumour samples (37). Based on the characteristic of controllable dissociation of metal-ligand coordination bonds under acidic conditions, Xia et al. further incorporated ICIs into such systems to construct pH-responsive synergistic antitumor nanoplatforms. They encapsulated Mn^2+^-siPD-L1 nanoprecipitate cores within a hyaluronic acid (HA) shell (AHA@MnP/siRNA). This structure not only effectively prevents drug degradation and leakage but also achieves tumour targeting through the binding of HA to the CD44 receptors on the surface of tumour cells. Compared to uncoated nanoprecipitates, AHA@MnP/siRNA exhibited significantly higher accumulation at tumour sites than in other normal tissues while prolonging drug circulation time. In the 4T1 model, AHA@MnP/siRNA demonstrated markedly enhanced antitumor efficacy by activating the STING pathway and downregulating Programmed cell death ligand 1 (PD-L1) expression in tumour cells (38). PD-L1 is an immune checkpoint protein expressed on tumour cells. It binds to the programmed death receptor-1 (PD-1) on T cells, thereby inhibiting the cytotoxicity of CD8^+^ T cells. Li et al. synthesised MnSe_2_-lipid nanoplatforms, which was designed to enhance radiotherapy sensitivity and mitigate radiotherapy-associated side effects simultaneously. This nanoplatform enhances tumour site enrichment and biocompatibility through lipid surface modification, while achieving pH responsiveness in the TME by forming metal-phenol coordination bonds between octadecyl gallate and MnSe_2_. In oesophageal squamous cell carcinoma models, Mn^2+^ released from MnSe_2_-lipid activates the STING pathway and significantly enhances tumour cell sensitivity to radiotherapy. Concurrently, Se’s antioxidant properties mitigate radiation-induced damage in normal tissues, achieving a dual effect that strengthens radiotherapy efficacy and provides radiation protection (39). Another study constructed a nanoparticle core formed by the supramolecular assembly of Fe^3+^, tannic acid, and zoledronic acid. This system achieves pH responsiveness through noncovalent interactions and metal coordination bonds. Subsequently, the nanoparticle surface was further modified with fucoidan to achieve targeted delivery to invasive and metastatic tumours. In the acidic TME, tannic acid reduces Fe^3+^ to Fe^2+^, catalyzing the generation of ROS from H_2_O_2_ via the Fenton reaction and triggering ferritin autophagy. Zoledronic acid further promotes ROS-mediated cytotoxicity and lipid peroxidation by inhibiting antioxidant enzymes, thereby enhancing ferroptosis and inducing mtDNA damage. Subsequently, the released mtDNA activates the STING pathway, initiating an anti-tumor immune response. In a 4T1 model, the nanoparticle exhibited high stability under physiological conditions while specifically accumulating in tumour tissues. It rapidly degrades within the TME to release the drug, synergistically inducing ferroptosis while activating the STING pathway, thereby inhibiting tumour growth and suppressing distant metastasis (40).

### 3.2 pH-responsive inorganic nanoparticle platform

Inorganic nanoparticles demonstrate broad application prospects in drug delivery due to their outstanding chemical stability, biocompatibility, and biomimetic properties. Particularly within the acidic TME, these nanoparticles exhibit significant pH responsiveness (41). For example, Xu et al. developed Mn-doped CaCO_3_ nanoparticles for the delivery of azacitidine, further stabilising them with polyethene glycol (PEG) to enhance biocompatibility. In the acidic TME, CaCO_3_ not only serves as a carrier for drug release but also enhances the bioactivity of Mn^2+^ through the released Ca^2+^. Intracellular Ca^2+^ accumulation triggers Ca^2+^ overload and synergistically generates substantial ROS with Mn^2+^, activating caspase-3. Concurrently, azacitidine upregulates GSDME expression and inhibits DNA methylation, further promoting caspase-3/GSDME-mediated pyroptosis. The resulting pyroptotic damage releases large amounts of dsDNA, activating the Mn^2+^-sensitive STING pathway. In the 4T1 mouse model, these nanoparticles achieved specific accumulation at tumour sites and effectively inhibited tumour growth, whereas azacitidine alone failed to produce significant antitumor effects. When combined with radiotherapy, this nanoparticle platform potently activated the STING pathway, thereby reshaping the immunosuppressive TME and significantly enhancing the therapeutic efficacy of radiotherapy (42). Hydroxyapatite (HAP), as the primary component of bone, exhibits good stability under physiological conditions but undergoes significant dissolution at pH values below 5.0. Based on the doping properties of HAP and its high affinity for risedronate (Ris), Zhang et al. developed Mn-doped, Ris-loaded nanoplatforms (MnHARis). *In vivo* experiments demonstrated that MnHARis exhibits excellent long-term stability and good biocompatibility. More importantly, the incorporation of Ris significantly enhanced the cancer immune response mediated by the Mn^2+^-triggered STING pathway (43). Another study reported a co-delivery system based on HAP nanoparticles for curcumin and L-oxaliplatin (L-OHP). This system exhibited high stability under physiological conditions, with release rates of 66.5%, 65.5%, and 60% for curcumin, L-OHP, and Ca^2+^, respectively, at pH 6.0. This nanoplatform induced substantial Ca^2+^ accumulation within tumour cells, while curcumin inhibited Ca^2+^ efflux, resulting in Ca^2+^ overload and mtDNA damage. L-OHP further causes nuclear DNA damage, and its synergistic action significantly activates the STING pathway. Compared to single-drug-loaded nanosystems, this co-delivery platform more effectively reverses the immunosuppressive properties of the colorectal cancer TME and exhibits more vigorous tumour growth inhibitory activity (44).

Mesoporous silica nanoparticles (MSNs) represent an emerging class of pH-responsive inorganic nanoparticles characterised by tunable pore sizes and large specific surface areas. They enable efficient drug loading while exhibiting excellent biocompatibility (45). Zhao et al. designed manganese-doped MSNs (M-MSNs) for delivering the antigen ovalbumen (OVA). These nanoparticles were further coated with mannose-modified bacterial cell membranes, enabling them to specifically recognise and bind to mannose receptors on the surface of DCs. In tumour-bearing mouse models, compared to non-mannose-modified nanoparticles, mannosyl-modified M-MSNs exhibited significantly greater enrichment in DCs and lymph nodes, demonstrating superior targeted delivery efficiency. Simultaneously, they showed more pronounced tumour growth inhibition and significantly prolonged the survival of mice. Crucially, beyond activating the STING pathway, the released Mn^2+^ also serves as a magnetic resonance imaging (MRI) contrast agent, enabling visual monitoring of the drug delivery process (46). MSNs can serve as delivery vehicles for the STING agonist cdGMP (immune MSN). Bielecki et al. modified MSN surfaces with primary and secondary amines to induce protonation under acidic conditions. This weakens the interaction forces between cGMP and MSNs, facilitating the targeted release of cGMP at tumour sites. *In vivo* experiments demonstrated that immune MSNs efficiently and specifically delivered cGMP to APCs within the TME, thereby inhibiting tumour growth. In a glioblastoma model, immune MSN treatment resulted in complete tumour clearance in 50% of mice, whereas tumours continued to grow in the blank MSN control group (47). Furthermore, numerous studies have demonstrated that Mn-based pH-responsive inorganic nanoparticles—such as MnCO_3_, mHMnO_4_, and MnO_2_—exhibit exceptional potential in tumour immunotherapy. These nanoparticles not only exhibit outstanding acid responsiveness but also demonstrate excellent tumour-targeting capabilities. They can effectively activate the STING pathway, thereby enhancing antitumor immune responses (48–50). For example, combining gold nanoparticles (Au NPs) with MnCO_3_ enables the construction of a MnCO_3_–Au composite nanoplatform. In this system, Au NPs catalyse the oxidation of glucose into gluconic acid and H_2_O_2_. The resulting acidic environment, combined with the acidic conditions of the TME, significantly promotes MnCO_3_ degradation, releasing Mn^2+^ and HCO_3_^-^. Subsequently, Mn^2+^ and HCO_3_^-^ further catalyse H_2_O_2_ to generate ROS, significantly enhancing Mn^2+^-mediated STING pathway activation. In mouse models, this composite nanoplatform demonstrated superior CD8^+^ T cell infiltration and tumour retention capabilities compared to MnCO_3_ or Au NPs alone, thereby achieving more substantial tumour suppression effects (51).

### 3.3 pH-responsive lipid nanoparticle platforms

Lipid nanoparticles (LNPs) are currently one of the most widely used drug delivery platforms, garnering significant attention due to their excellent biocompatibility, biodegradability, and high encapsulation efficiency of drugs. As a typical amphiphilic structure, LNPs can simultaneously load both hydrophilic and hydrophobic drugs, thereby mimicking natural cell membranes and significantly enhancing cellular uptake and endocytosis processes (45). Based on this, Xian et al. incorporated piperazine into the MSA-2 molecule, enabling it not only to encapsulate within pH-responsive LNPs stably but also to facilitate prodrug transport and release into the cytoplasm via piperazine protonation under acidic TMEs. This design achieves a “dual-switch” regulatory mechanism: first enabling tumour-targeted accumulation *in vivo*, followed by efficient intracellular drug release. Compared to traditional liposomes, this nanoplatform exhibits a longer circulation time, higher tumour delivery efficiency, and improved tumour targeting ability within the circulatory system. In a triple-negative breast cancer (TNBC) model, the platform significantly activated the STING pathway, thereby inducing a robust antitumor immune response (52). In another study, researchers further engineered pH-responsive LNPs (pLCGM-OVA) by modifying the platelet-derived growth factor B cyclic peptide, endowing them with active tumour-targeting capabilities. This nanoplatform induces cuproptosis by delivering CuGdMn nanoclusters and OVA, thereby activating the STING pathway. Cuproptosis is similar to ferroptosis in that it primarily induces mitochondrial damage, leading to the release of mtDNA into the cytoplasm and activating the STING pathway. However, cuproptosis mainly depends on excess copper binding to lipoylated proteins, which interferes with iron-sulfur cluster proteins in the respiratory chain, resulting in mitochondrial dysfunction. Following systemic injection, pLCGM-OVA exhibited more than double the blood half-life compared to unmodified lipid nanoparticles, with significantly increased accumulation at tumour sites while maintaining excellent biocompatibility. In a melanoma model, pLCGM-OVA significantly promoted DCs maturation and cytotoxic T cell infiltration, effectively suppressing tumour growth and recurrence. Furthermore, the platform demonstrated outstanding MRI performance, enabling visual monitoring of drug delivery and therapeutic guidance (53).

### 3.4 pH-responsive metal-organic framework (MOF) nanoplatforms

Zeolitic imidazolate framework-8 (ZIF-8) is an emerging MOF whose characteristic pH responsiveness arises from the instability of metal coordination bonds and noncovalent interactions under acidic conditions, leading to the gradual degradation of its Zn^2+^-2-methylimidazole framework (54). Hu et al. encapsulated MnCO and doxorubicin (DOX) within ZIF-8 (ZMD). In the acidic TME, the degradation of ZIF-8 releases Mn^2+^, Zn^2+^, and DOX. These ions not only activate the STING pathway to trigger potent anti-tumour immune responses but also promote ROS production, thereby enhancing DOX-induced DNA damage and apoptosis in tumour cells. In mouse tumour models, ZMD reached peak concentrations at tumour sites within 8 h and was partially cleared after 24 h, causing no significant damage to the heart, liver, or spleen. More importantly, when combined with radiotherapy, ZMD significantly outperformed ZIF-8 and ZIF-8@MnCO in inhibiting tumour growth. This achieves synergistic effects between chemotherapy, immunotherapy, and radiotherapy (55). Another study loaded the COX-2 inhibitor C-phycocyanin (CPC) onto ZIF-8 and achieved active targeting to tumours overexpressing folate receptors through the use of folate-PEG-NH_2_ modification. The incorporation of CPC weakened the interaction between Zn^2+^ and 2-methylimidazole, accelerating acid-induced framework dissociation. It also suppressed COX-2 upregulation in tumour cells, reduced the accumulation of immunosuppressive cells, and further enhanced Zn^2+^-mediated STING pathway activation. In mouse tumour models, ZIF-8@FA-PEG-NH_2_@CPC demonstrated significant preferential accumulation in tumour tissues and rapidly disintegrated within the acidic environment of lysosomes. Compared to ZIF-8@FA-PEG-NH_2_, it released approximately 3.1 times more Zn^2+^ and induced stronger CD8^+^ T cell infiltration. Compared to ZIF-8 alone, this nanosystem induced approximately fourfold greater tumour regression (56).

### 3.5 pH-responsive polymers

Polymers are widely used as materials for constructing nanocarriers due to their tunable structures and stability under physiological conditions. Among them, polyacrylic acid is one of the most commonly used biodegradable polymers (57). For example, Shae et al. synthesised a pH-responsive copolymer STING-NPs, whose pH responsiveness stems from the reversible protonation of diethylaminoethyl methacrylate units within acidic endosomes. In mice, STING-NPs were well-tolerated, with no hepatotoxicity or nephrotoxicity, while promoting the accumulation of the loaded drug in lymph nodes, demonstrating excellent tumour localisation ability and biocompatibility. More importantly, STING-NPs enhanced the efficacy of cGAMP by 240–610-fold. In melanoma models, cGAMP-loaded STING-NPs reduced tumour growth rates approximately 11-fold compared to free cGAMP, significantly suppressed tumour recurrence, and exhibited potent inhibition of distant tumours—effects absent with free cGAMP (58). Furthermore, this copolymer can co-deliver peptide antigen (Pre) and monophosphoryl lipid A (MPLA), further enhancing cGAMP’s STING activation capacity. When combined with ICIs, cGAMP/pre/MPLA@STING-NPs significantly outperformed cGAMP/Pre@STING-NPs in prolonging mouse survival, promoting CD8^+^ T cell responses, and inhibiting tumour growth (59). Beyond serving as delivery systems, specific polymers possess immune-activating functions themselves. PC7A is an innovative pH-responsive polymer that combines carrier functionality with intrinsic STING activation capabilities. It induces innate immune responses by triggering STING-mediated aggregation. Compared to cGAMP, it prolongs cytokine expression. In tumour-bearing mouse models, cGAMP@PC7A significantly enhanced CD8^+^ T cell infiltration and sustained anti-tumour immune activation, effectively inhibiting tumour growth without observable toxicity. In contrast, free cGAMP or PC7A alone induced only minor immune responses (60). Importantly, PC7A can efficiently co-deliver STING agonists with other antitumor agents (such as DOX, PD-1/PD-L1-targeting peptides, and MPLA), enabling the reprogramming of the TME and establishment of long-term immune memory in models of pancreatic ductal adenocarcinoma and osteosarcoma (61, 62). Moreover, Wang et al. engineered polymeric nanoparticles centred on LaP–siRNA/cGAMP to achieve a multi-factor synergistic immune amplification mechanism. These nanoparticles achieve pH-responsive release through acid-sensitive dissolution of LaP and reversible protonation of the outer imidazole groups. Within this system, La^3+^ promotes STING activation, PTPN6-siRNA relieves STING inhibition, while cGAMP directly initiates the STING pathway as a ligand. Together, these components form a mutually amplifying, cascading positive-feedback immune activation network. In tumour-bearing mice, this nanoplatform induced systemic antitumor immunity and generated long-lasting antitumor memory, while also inhibiting the progression of distant tumours (63). Polyamino esters represent another widely used class of pH-sensitive polymers, characterised by excellent biocompatibility. Under acidic conditions, they undergo protonation, become hydrophilic, and disassemble. Liu et al. developed a poly(β-amino ester) material with pH-triggered self-degradation properties. This polymer undergoes primarily slow hydrolysis at pH 5.5 but rapidly self-degrades at pH levels above 6.5, enabling its swift disintegration within the TME and the release of degradation products. The resulting nanoparticle vaccine p(S+O) efficiently delivers OVA and 2′3′-cGAMP to the cytoplasm, significantly enhancing innate immune activation and reshaping the immunosuppressive TME, thereby inducing potent antitumor immune responses. Notably, p(S+O) also mitigates systemic inflammatory responses triggered by free 2′3′-cGAMP. In melanoma models, compared to free OVA + 2′3′-cGAMP, p(S+O) demonstrated significant advantages in lymph node activation, T cell responses, suppression of immunosuppressive cells, and antitumor efficacy (64).

In summary, pH-responsive STING-activating nanoplatforms can achieve precise drug release by leveraging the acidic TME and enable active targeting through receptor modification. **Table 1** summarises pH-responsive nanoplatforms used to activate the STING pathway. However, this strategy still faces several limitations. First, pH variations within tumours and across different tumour types may compromise the precision of drug release. Second, reduced acidity in diseased or inflamed tissues could induce off-target effects. Additionally, complex fabrication processes for certain nanoplatforms (e.g., polymer carriers) hinder large-scale production, while simplified methods, such as self-assembly, though convenient, often cannot directly load STING agonists, thereby limiting the efficiency of immune activation. Despite these challenges, pH-responsive STING-activating nanoplatforms remain a crucial foundation with significant developmental potential for cancer immunotherapy.

**Table 1:** pH-responsive nanodelivery systems for STING activation.

Nanoplatform	Nanoparticles	Content	Reference
Black phosphorus nanosheet	BPNS@Mn^2+^/CpG	Mn+CpG ODNs	(65)
CaCO_3_	Aza@Mn-CaCO_3_-PEG	Mn+Azacitidine+Ca	(42)
	CM NPs	Mn+pPHS	(66)
	M-CNP/Mn@pPHS	Mn+Curcumin+Ca	(67)
Hydroxyapatite	Cur/L‐OHP@HAP	Curcumin+L‐oxaliplatin	(44)
	CS-HAP@KAE NPs	CS+kaempferol	(68)
	MnHARis	Risedronate+Mn	(43)
Lipid nanoparticles	Nanoadjuvants	MSA-2	(52)
	MnSe_2_-lipid	Mn	(39)
	Nanovaccines	cGAMP+KRAS mRNA	(69)
	pLCGM-OVA	Mn+OVA	(53)
	ALFM	FePt+Mn	(70)
Layered double hydroxide	BSA-LDHs-cGAMP	cGAMP	(71)
	IFNγ/uMn-LDHs	Mn+IFNγ	(72)
Manganese phosphate nanoparticles	AHA@MnP/siPD‐L1	Mn+siPD‐L1	(38)
Mn-enriched Zn peroxide nanoparticles	MOMP	Mn+Zn	(73)
Mesoporous silica	OVA@MMSNs@BM-Man	Mn+OVA	(46)
	Immuno-MSN	cdGMP	(47)
	MnO@mSiO-iRGD 2 NPs	MnO+RGD	(74)
MnCO_3_	MnCO_3_–Au	Mn	(51)
	Mn_1–x_Fe_x_CO_3_	Mn+OVA+Fe	(50)
MnP	OVA@MnP	Mn	(75)
mHMnO	mHMnO-Dox	DOX+Mn	(48)
MnO_2_	αPD-L1@MnO_2_	αPD-L1+Mn	(49)
	PTC209/MnO_2_@BSA	Mn+PTC209	(76)
Prussian blue	PB–Mn/OVA NE	Mn+OVA	(77)
Polydopamine nanoparticles	PDA-Mn-HA NPs	Mn	(78)
	Cu-ZnO_2_@PDA	Cu+Zn	(79)
Polymersomes	CQCP	Chloroquine+CDA	(62)
	IAHA-LaP/siPTPN6 NPs	siPTPN6+La^3+^+cGAMP	(63)
	Nanoparticle platform	Peptide+cGAMP+MPLA	(59)
	Multi-component NPs	DOX+MPLA+PLTP	(61)
	D-SAM	cGAMP	(80)
	PC6AB@MnP	MnP	(81)
	M-PNP@R@C	Resiquimod+cGAMP	(82)
	dPEDE-A@M32	ADU-S100+Mannose	(83)
	nPGSA	cGAMP	(84)
	NanoISD	dsDNA	(85)
	PMM NPs	Mn_34_+MPLA	(86)
	PLHM-DOX NPs	DOX+Mn	(87)
	PEIM@OVA	MSA-2+OVA	(88)
Phospholipid bilayer shell	NanoMn-GOx-PTX	GOx+PTX+Mn	(89)
Self-assembled nanoparticles	FANP	Raddeanin A+Fe	(37)
ZnS	ZnS@BSA Nanoclusters	Zn	(90)
Zinc–Mn–CDN Particle	ZMCP	CDA+Mn	(91)
Zoledronic acid nanoparticles	FTZ@Fu SANs	Zoledronic acid	(40)
Zeolite imidazole framework 8	ZIF-8@MnCO@DOX	MnCO+DOX	(55)
	RIZM NPs	Rapamycin+ICG	(54)
	CZFNPs	C-phycocyanin	(56)

DOX, doxorubicin; ICG, indocyanine green; CDA, cyclic di-AMP; D-SAM, dual-STING-activating micelle system; MPLA, monophosphoryl lipid A; OVA, ovalbumin; iRGD, tumour homing peptide; PTC209, B-cell-specific Moloney murine leukemia virus insertion site 1 inhibitor; CS, Chondroitin sulfate; PLTP, PD-1/PD-L1-targeting peptide; Gox, glucose oxidase; PTX, paclitaxel; CpG ODNs, CpG oligodeoxynucleotides; pPHS, pyroptosis-inducing plasmid.

## 4 Redox-responsive nanoplatforms

Redox homeostasis not only maintains intracellular environmental balance but also functions as a critical component of signalling networks through reversible regulatory mechanisms, mediating various physiological responses. However, under the influence of factors such as hypoxia, mitochondrial metabolic alterations, oncogene activation, and low pH, cancer cells produce higher levels of ROS than normal cells, leading to a TME with elevated ROS (92). Concurrently, increased ROS levels induce compensatory activation of antioxidant systems, such as the upregulation of GSH, to maintain redox homeostasis (2). Therefore, both ROS and GSH are typical features of the TME. Although these features pose significant challenges for cancer therapy, redox-responsive nanocarriers designed based on them can not only achieve precise spatiotemporal drug release but also modulate ROS and GSH levels within the TME, thereby enhancing the sensitivity of cancer cells. **Table 2** summarises redox-responsive nanoplatforms for STING activation.

**Table 2:** Redox-responsive nanoplatforms for STING activation.

Responsive moiety	Nanoplatform	Nanoparticles	Content	Reference
ROS-responsive nanodelivery systems				
Thioketal linkages	Polymersomes	NPMn/NPPt	Mn/CisPt	(93)
	Polymersomes	NPs	Camptothecin+CisPt	(94)
	Polymersomes	NP–Pt-IDOi	NLG919+CisPt	(95)
	Lipid nanoparticles	Lipo/TK-CDN/TPP/Ce6	Cyclic dinucleotide+chlorin E6	(96)
Boronic ester bond	Polymersomes	DPGMA	Gossypol+Mn+anti-PD-L1	(97)
GSH-responsive nanodelivery systems				
Disulfide bond	mPEG-Pglu (ss-ER)	iPR@M1/Se	LND+Se+P-gp+iRGD	(98)
	Mesoporous silica	PF/MMSN@MPM	CHK1+Mn	(99)
	Mesoporous silica	dsDNA@DMONs	dsDNA	(100)
Disulfide bond	Polymersomes	NP2	CisPt+WEE1 inhibitor	(101)
	Polymersomes	—	MSA-2 cobalt(III) cyclam	(102)
	Polymersomes	NP(3S)	CisPt	(103)
	HMON	Fe^0^@HMON@DNA-Exo	Fe	(104)
Diselenide bonds	MSPs	RDPNs@diABZIs	diABZIs+MeβCD	(105)
	Self-assembled nanoparticles	MN NPs	NLG919+MSA-2	(106)
Coordination bond	Polydopamine	MnPDA	Mn	(107)
	Metal-organic nano-frameworks	Cu-MOF@CDDP	CisPt	(108)
	ZnFe_2_O_4_	ZnFe_2_O_4_-PTX	Zn+Fe+PTX	(109)
Mn-O	MnO_2_	MGPP NPs	Mn+cGAMP+GOx	(110)
	MnO_2_	OV-MnO_2_/HE	Mn+Oncolytic viruses	(111)
	MnO_2_	MnO_2_@APS-IR820	Squamocin+Mn	(112)
	MnO_2_	Mn-MC NP	Mn+chlorin e6+MSA-2	(113)
	Metal-organic nano-frameworks	MnxOy/(A/R)TiO_2_	Mn	(114)
	MMONs	TPP-MMONs	Mn	(115)

LND, lonidamine; Senaparib, Se(Poly[ADP-ribose] polymerase inhibitor); HMON, hollow mesoporous organosilica nanoparticles; MMONs, manganese dioxide-doped mesoporous organosilica nanoparticles; TPP, Triphenyl-phosphine; CHK1, checkpoint kinase; CDN, cyclic dinucleotide; HA, Hyaluronic acid; PMM, macrophage membranes expressing Pep20, MMP2, and M2pep; GelMA, photo-cross-linked methacrylated gelatin; MMF, monomethyl fumarate; HACS NPs, hypoxia-responsive, crosslinked albumin-based nanoparticles; DPPC, 1,2-dipalmitoyl-sn-glycero-3-phosphocholine; ECM-DNAs, extracellular matrix-degrading nanoagonist; OMVs, *escherichia coli*-derived outer membrane vesicles; EM, exosome-mimetic; AMCM, antigen-enriched cell membrane.

### 4.1 ROS-responsive nanoplatforms

Boric acid esters, thioketals, and thioethers are the most widely studied ROS-responsive moieties. Their low toxicity and high chemical stability make them ideal choices for constructing ROS-responsive nanoscale platforms (36). For example, Cao et al. designed a dual ROS-responsive nanoplatform. First, they engineered cisplatin and the topoisomerase I inhibitor camptothecin (CPT) into the platinum(IV) prodrug CPT-Pt(IV). This allows it to be reduced by ROS within tumour cells, releasing active cisplatin and CPT. Subsequently, CPT-Pt(IV) self-assembled with a thioketal-containing polymer to form nanoparticles. In mice, these nanoparticles accumulated at tumour sites in a time-dependent manner with negligible systemic toxicity. In colorectal cancer models, the two drugs synergistically induced apoptosis and dsDNA breaks, thereby activating the STING pathway and generating a strong anti-tumour immune response (94). Further studies demonstrated that this nanocarrier can also self-assemble with a novel manganese complex (TPA-Mn) to form NPMn nanoparticles. In ovarian cancer peritoneal metastasis and recurrence models, NPMn nanoparticles combined with anti-PD-1 antibodies and cisplatin exerted potent inhibitory effects on both primary and recurrent tumours by activating the STING pathway. More importantly, this nanoplatform achieved long-term immune protection by reshaping the immunosuppressive TME (93). Moreover, Wang et al. constructed a nanocomposite (DPGMA) capable of delivering Mn^2+^ and anti-PD-L1 antibodies by coupling polymers with gossypol (Gos) via borate ester bonds. Concurrently, the coordination bond formed between Mn^2+^ and Gos endowed this nanoplatform with pH responsiveness. In mice, DPGMA demonstrated excellent water solubility and effectively targeted tumour sites. Notably, Gos synergised with Mn^2+^ and anti-PD-L1 to exert anti-tumour immune effects by inhibiting tumour cell proliferation and inducing ROS generation, achieving sustained and significant tumour suppression in a melanoma model (97).

### 4.2 GSH-responsive nanoplatforms

Incorporating disulfide or diselenide bonds as linkages is a widely used strategy for constructing GSH-responsive nanodelivery systems. These bonds can rapidly cleave under high GSH concentrations, thereby triggering the degradation of nanocarriers or the dissociation between prodrugs and their carriers (116). Zhu et al. constructed a disulfide-based GSH-responsive nanoplatform integrating the tumour-targeting peptide iRGD and the P-gp inhibitor ER. This nanoplatform was employed for the synergistic delivery of the lonidamine prodrug (LND) and senaparib (Se). In the TME, iRGD targets αvβ3 integrins on tumour cell surfaces, enabling selective accumulation of the nanoparticles in tumours. GSH subsequently induces nanoparticle disassembly and drug release, while ER enhances the intracellular accumulation of LND and Se, synergistically promoting DNA damage and activating the STING pathway. Compared with Se monotherapy, this strategy not only reverses drug resistance in TNBC but also induces durable immune memory, resulting in significantly enhanced tumour inhibition (98). Moreover, the depletion of GSH by GSH-responsive nanocarriers can improve the efficacy of ROS-based cancer therapies. Recently, a trisulfide bond-containing nanoplatform (NPs-3S) was developed for the delivery of cisplatin. NPs-3S efficiently depletes GSH in the TME and releases H_2_S, thereby elevating ROS levels to potentiate cisplatin-induced cell death and DNA damage. *In vivo* experiments demonstrate that NPs-3S significantly accumulates in tumour tissues and potently activates the STING pathway, thereby promoting the infiltration and activation of CD8^+^ T cells. Additionally, NPs-3S markedly reduces cisplatin-associated hepatotoxicity (103). Guo et al. developed hollow mesoporous organic silica nanoparticles responsive to GSH. They further modified these nanoparticles by using tumour cell membranes, endowing the nanoplatform with specific homing capability toward TNBC cells. *In vivo*, this nanomedicine releases Fe^0^ within the high-GSH TME, where it reacts with GSH to induce ferroptosis. This process generates a ROS storm that effectively suppresses tumour growth. Crucially, the platform also precisely activates the STING pathway within APCs (104). Compared with disulfide bonds, diselenide bonds exhibit greater responsiveness to various redox environments. Luo et al. constructed a GSH-responsive supramolecular polycyclodextrin containing diselenium bonds for delivering methyl-β-cyclodextrin (MeβCD) and the STING agonist diABZIs (RDPNs@diABZIs). RDPNs@diABZIs enable the simultaneous release of MeβCD and diABZIs in the reductive TME. The released MeβCD depletes membrane cholesterol, suppressing tumour cell softening and thereby blocking biomechanics-mediated immune inhibitory pathways, which enhances CTL-mediated killing of cancer cells by diABZIs. *In vivo* experiments demonstrate that compared to free diABZIs, RDPNs@diABZIs not only significantly eliminate tumours and induce persistent immune memory, but also exhibit long-term retention and selective accumulation at tumour sites (105). Additionally, GSH can achieve drug release through competitive coordination with metal ions. Duan et al. combined Cu^2+^ and the IDO inhibitor NLG919 with MSA-2 to form nanoparticles through self-assembly. In a melanoma model, GSH competes with Cu^2+^, triggering the rapid release of NLG919 and MSA-2, and thereby inducing a robust immune response that effectively suppresses both primary and metastatic tumours (106).

Taken together, redox-responsive nanoplatforms achieve precise and controllable drug release by leveraging ROS and GSH in the TME. They can also amplify immunogenic signals such as DNA damage, ferroptosis, or metal ion stimulation by modulating redox homeostasis, thereby effectively enhancing STING pathway activation and subsequent antitumour immune responses. However, similar to pH-responsive nanoplatforms, the heterogeneity of redox levels and dynamic fluctuations in ROS/GSH concentrations within the TME may compromise drug release precision and induce off-target effects. Furthermore, excessive reliance on redox regulation risks causing non-specific oxidative damage in normal tissues, increasing potential safety concerns.

## 5 Enzyme-responsive nanoplatforms

Enzymes play a critical regulatory role in biological metabolism. However, in the TME, enzymes such as matrix metalloproteinases (MMPs) and cathepsin B are often overexpressed, and this aberrant activity promotes tumour cell invasion and metastasis. Therefore, enzyme-responsive nanocarriers can leverage the specificity of enzymes within the TME to achieve site-specific drug release (36) (**Table 3**). Miao et al. constructed a human serum albumin (HSA) nanoplatform (SH-NPs) loaded with SR-717 based on the degradation characteristics of cathepsin B toward HSA. Under physiological conditions, SH-NPs effectively prevented the premature release of SR-717, while free SR-717 release reached up to 90%. Furthermore, compared with SR-717, long-term use of SH-NPs did not increase organ toxicity or immunogenicity. Tumour-bearing mouse experiments demonstrated that SH-NPs accumulated explicitly at tumour sites, inducing STING activation at a level twice that of free SR-717. When combined with anti-PD-L1 antibodies, SH-NPs significantly delayed tumour growth and improved survival rates. These findings demonstrate that SH-NPs mitigate the toxicity risks and off-target responses associated with SR-717, while exhibiting excellent biocompatibility and tumour-targeting capabilities (137). In another study, enzyme-responsive poly(β-amino ester) nanoplatforms can not only target tumour sites but also be internalised by tumour cells, enabling the gradual release of CDN drugs over time. This allows sustained activation of the STING pathway and promotes the formation of immune memory, significantly inhibiting tumour growth and reducing the risk of recurrence (138). MMPs are a whole family of enzymes that degrade a wide variety of proteins in the extracellular matrix. Hussain et al. designed an innovative nanoparticle system that loads STING agonists into a porous zirconium-based metal-organic framework. The system was further functionalized with cell membrane expressing Pep20, an MMP2, and macrophage-targeting peptide (M2pep) (ZrMOF/C@P). In the TME, the PLGLAG fragment within the MMP-2 substrate sequence on the cell membrane is cleaved by MMPs, triggering Pep20 release and blocking the CD47/SIRPα pathway, thereby restoring macrophage phagocytic activity (140). CD47 is a tumour-specific “don’t eat me” signal that interacts with signal regulatory protein α (SIRPα) on macrophages, thereby inhibiting macrophage-mediated phagocytosis. Subsequently, guided by the M2pep, the nanoparticles are internalised by M2-like macrophages, releasing the STING agonist and activating the STING signalling pathway. In the T41 model, ZrMOF/C@P exhibited significant accumulation at the tumour site even 72 h post-injection, enabling sustained inhibition of tumour growth (140). In fact, a notable advantage of enzyme-responsive nanoplatforms is their ability to prolong the circulation time of the drug significantly. For example, Jiang et al. developed a hydrogel-based nanoplatform for puncture-needle delivery of Cu_0.5_Mn_2.5_O_4_ nanoparticles. *In vivo*, this nanoplatform achieved sustained drug release for at least 15 days through the action of MMPs (141). In addition, enzymes such as esterases, Hyaluronidases, and caspases are also frequently employed in the design of enzyme-responsive nanodelivery platforms (142). For example, in an HA-conjugated MnFe_2_O_4_@NaGdF_4_ nanoplatform, the intrinsic tumour-targeting capability of HA, together with its specific degradation by hyaluronidase, enables efficient tumour accumulation and drug release in tumour regions (139). In short, enzyme-responsive nanoplatforms exhibit excellent tumour-targeting ability, sustained drug release, and safety. However, because enzymatic kinetic reactions are influenced by factors such as enzyme content and reaction environment, the drug release performance of these nanoplatforms may vary significantly among individuals. This increases the difficulty of determining appropriate dosages in clinical settings. Additionally, their design and preparation are relatively complex, further limiting their application. Therefore, more in-depth research is needed to optimise their clinical feasibility.

**Table 3:** Other stimuli-responsive nanoplatforms for STING activation.

Responsive moiety	Nanoplatform	Nanoparticles	Content	Reference
Hypoxia-responsive nanodelivery systems				
Azobenzene	Human serum albumin	HACS NPs	AQ4N+SR-717+CaCO_3_	(117)
Light-responsive nanodelivery systems				
Chlorin e6	Self-assembled nanoparticles	TPP-Ce6@siPD-L1	siPD-L1	(118)
	Hollow silica nanoparticles	Pt@RHC-LXL-1/CLP002	Peptide CLP002+Pt	(119)
DPPC	Lipid nanoparticles	ECM-DNAs	Bromelain+FeS_2_+cGAMP	(120)
Pyropheophorbide-a	Peptide	SNVac	STING agonist	(121)
Photodynamic monomer	Polymersomes	NP^PDT^-56MESS	Platinum drug 56MESS	(122)
Indocyanine green	Self-assembled nanoparticles	Co+diABZI@J-dICG	Co+diABZI	(123)
Perfluoropentane	Lipid nanoparticles	ODVLipo/SPIO-CREKA	SPIO+cGAMP+CREKA	(124)
Pluronic	Polymersomes	NvIH	ICI antibodies+cGAMP	(125)
Ultrasound-responsive nanodelivery systems				
Chlorin e6	Lipid nanoparticles	RCM-Lip	MSA-2	(126)
	Lipid nanoparticles	Ce6/PTX Nbs	Paclitaxel	(127)
Magnetic field-responsive nanodelivery systems				
Fe_34_	—	Fe_3_O_4_@Ca/MnCO_3_	Ca+Mn+Tumour antigen	(128)
Cell membrane-responsive nanodelivery systems				
AMCM	—	AECM@PC7A	PC7A	(129)
OMVs	—	OMV/SaFeFA	Fe^2+^+STING agonist-4	(130)
Tumour cell membrane	—	EM@REV@DOX	REV+DOX	(131)
	—	cGAMP@vEVs	cGAMP	(132)
	—	PLGA/STING@EPBM	PLGA/STING agonist	(133)
Hybrid cell membrane	—	hNV	cGAMP	(134)
EM-nanocarriers	—	EM@REV@DOX	DOX+REV	(131)
Radiation-responsive nanodelivery systems				
Zeolite imidazole framework 8	Zeolite imidazole framework 8	Mn-ZIF-8	Mn	(135)
Diselenide	Au NPs	sMnAu NAs	Au	(136)
Enzyme-responsive nanodelivery system				
Cathepsin B	Human serum albumin	SH-NPs	STING agonist SR-717	(137)
	Poly(β-amino ester)	—	Cyclic dinucleotide	(138)
Hyaluronidase	MnFe_2_O_4_@NaGdF_4_	MGNH	NLG919+HA+Mn	(139)
Matrix Metallopeptidase 2	zirconium-based metal-organic framework	ZrMOF/C@PMM	PMM+cGAMP	(140)
	GelMA	Cu_0.5_Mn_2.5_O_4_	MMF+Mn	(141)

## 6 Hypoxia-responsive nanoplatforms

Given the limited sensitivity of early-stage tumours to pH and enzyme-responsive strategies, coupled with hypoxia being a persistent feature throughout all stages of tumour development, hypoxia-responsive nanoplatforms have emerged as a key strategy for achieving precise drug delivery (143). Based on changes in tumour hypoxia levels following high-intensity focused ultrasound (HIFU) therapy, a novel HSA-azobenzene crosslinked nanomedicine system (HACS) has recently been developed. Leveraging HACS’s superior photoacoustic imaging capabilities, sustained *in vivo* tumour targeting, and high accumulation efficiency, this system significantly enhances the guidance efficacy of HIFU procedures by improving imaging precision and accuracy. HIFU ablation induces a pronounced hypoxic microenvironment, triggering the release of ICD inducers, including anthraquinone 4-nitro (AQ4N), SR-717, and CaCO_3_. Subsequently, CaCO_3_ consumes lactate to alleviate immunosuppression, further supporting AQ4N- and SR-717-mediated ICD, STING activation, and immune cell infiltration, thereby eliminating residual tumour foci post-surgery. This system not only promoted CD8^+^ T cell infiltration within tumours but also enhanced NK cell activation and function through STING pathway activation. In the 4T1 tumour model, HACS demonstrated superior suppression of both primary tumours and distant metastases compared to the SR-717-unloaded control group, with 90% mouse survival maintained at day 30 (117). Taken together, the hypoxia-responsive nanoplatform offers a versatile and efficient strategy that combines precise imaging with STING agonists, thereby enhancing therapeutic targeting and antitumour efficacy.

## 7 Cell membrane-responsive nanoplatforms

Cell membrane-responsive nanoplatforms represent an emerging class of drug delivery systems. By encapsulating cell membranes from diverse sources onto nanoparticle cores, these carriers exhibit enhanced targeted delivery properties and superior biocompatibility (144). Outer membrane vesicles (OMVs) are nanoscale vesicles spontaneously released by Gram-negative bacteria. Sun et al. constructed a folate-modified OMVs nanoplatform (OMV/Sa-Fe-FA) for delivering Fe^2+^ and STING agonist-4. In a colon cancer mouse model, OMV/Sa-Fe-FA significantly enhanced tumour weight inhibition rate (77.6%), markedly outperforming the OMV/FA group, which exhibited only mild inhibitory effects. Furthermore, compared to the untreated group, no significant abnormalities were observed in histological and blood biochemical indicators of major organs after treatment with this nanoplatform. These results indicate that OMVs not only enhance the antitumour efficacy and tumour targeting of STING agonists but also exhibit excellent biocompatibility (130). In addition to membrane materials not derived from cells, membrane carriers originating from living cells—such as red blood cell membranes, tumour cell membranes, and white blood cell membranes—have also been widely utilised to construct multifunctional nanoplatforms (145). Guo et al. developed a nanovesicle derived from lung cancer cell membranes for the co-delivery of DOX and the STING agonist REV (EM@REV@DOX). Leveraging the tumour retention and targeting properties conferred by the cancer cell membrane, EM@REV@DOX is taken up by tumour cells in a time- and dose-dependent manner. It gradually degrades within the acidic TME, releasing DOX and REV to achieve efficient tumour accumulation and prolonged retention. In a lung cancer mouse model, compared to EM@DOX, EM@REV@DOX significantly increased the infiltration of CD8^+^ cytotoxic T cells in tumour tissues, exerting stronger suppression on the primary tumour. Crucially, no significant differences were observed in liver and kidney function or blood counts between EM@REV@DOX-treated and control mice, demonstrating excellent biosafety (131). Furthermore, this cell membrane-derived nanoplatform can be genetically engineered to enhance its tumour targeting capabilities. Wang et al. infected mouse 4T1 cells with vesicular stomatitis virus (VSV) to generate nanovesicles displaying VSV glycoprotein (VSVG) and calreticulin (CRT) on the membrane surface. Through the specific recognition of CRT by DCs and the tumour-homing properties, cGAMP@vEVs efficiently accumulated in lymph nodes and tumours. In the acidic environment of endosomes or the TME, VSVG triggers membrane fusion between target cells and cGAMP@vEVs, thereby bypassing endocytic uptake and releasing cGAMP. In a lung metastasis model, treatment with these nanoparticles significantly suppressed tumour metastasis, whereas the free cGAMP group exhibited the opposite effect (132). Overall, cell membrane-responsive nanoplatforms offer an excellent carrier option for STING agonist delivery due to their favourable biocompatibility and tumour-targeting capabilities (**Table 3**).

## 8 Light-responsive nanoplatforms

In addition to endogenous triggers, exogenous stimuli such as lasers, ultrasound, and X-rays can also induce site-specific degradation of nanocarriers. Among them, light is especially promising in nanomedicine due to its clinical accessibility, noninvasiveness, and precise controllability (146). Chlorin e6 (Ce6) and indocyanine green (ICG) are the most commonly used linkers for constructing photoresponsive nanocarriers, and are also photosensitizers for photodynamic therapy (PDT) and photothermal therapy (PTT). Therefore, the nanoplatforms built based on them not only have photoresponsive properties, but can also work synergistically with PDT and photothermal therapy PTT, thereby enhancing the anti-tumour efficacy (147) (**Table 3**). Luo et al. developed a TPP-Ce6@siPD-L1 nanoplatform self-assembled from Ce6 modified with the mitochondrial-targeting molecule triphenylphosphonium (TPP) (Ce6-TPP) and siPD-L1. Upon internalisation by tumour cells, this nanoplatform generates ROS under near-infrared (NIR) light irradiation, promoting its escape and accumulation within mitochondria. Subsequently, ROS induces mitochondrial dysfunction and apoptosis, releasing dsDNA to activate the STING pathway. This synergises with siPD-L1 to generate potent anti-cancer immune responses. In mouse models of tumourigenesis, TPP-Ce6@siPD-L1 demonstrated favourable safety and achieved an 82% tumour inhibition rate, significantly outperforming TPP-Ce6 (33%) (118). Beyond its role as a photosensitizer, Ce6 also functions as an ultrasensitizer. Yang et al. developed a lipid nanoparticle (RCM-Lip) loaded with Ce6, MSA-2, and iRGD to achieve ultrasound-triggered controlled drug release. In tumour-bearing mice, RCM-Lip significantly enhanced tumour-specific accumulation and local release efficiency due to iRGD targeting and ultrasound-mediated stimulation response. This effectively reduced off-target inflammation and autoimmune-like reactions induced by MSA-2. Notably, RCM-Lip achieved a tumour inhibition rate of 73.5%, in stark contrast to the non-MSA-2-loaded nanoplatform, which failed to suppress tumour growth. Crucially, no significant antitumour effect was observed without ultrasound stimulation (126).

In contrast, due to ICG’s higher photothermal conversion efficiency, ICG-based nanosystems are more commonly used in PTT. Recent studies have reported the self-assembly of a Co+diABZI@J-dICG nanoparticle from dimerised ICG (J-dICG), Co, and diABZI. Co+diABZI@J-dICG can generate localised high temperatures at tumour sites, directly inducing tumour cell death. Simultaneously, the synergistic interaction between Co and diABZI activates the STING pathway, thereby enhancing antitumour immune responses and significantly improving overall therapeutic efficacy. In a hepatocellular carcinoma model, the Co+diABZI@J-dICG group exhibited the smallest tumour foci post-treatment and significantly suppressed tumour recurrence, demonstrating markedly superior efficacy compared to the diABZI@J-dICG or J-dICG+NIR groups. Additionally, ICG possesses a natural affinity for liver accumulation, which facilitates sustained retention of this nanoplatform at tumour sites, thereby reducing its non-specific distribution throughout the body (123). Furthermore, certain nanoplatforms can leverage the inherent properties of the carrier itself to achieve photothermal conversion, thereby enabling their use in PTT. Recently reported Mn-doped Prussian blue (PB-Mn/OVA NEs), Mn-doped polydopamine nanoparticles, and Au@MnO_2_ nanoplatforms have all demonstrated outstanding photothermal performance (77, 107, 148). For example, in tumour-bearing mice, PB-Mn/OVA NEs elevated the temperature of the tumour region to 60 °C under NIR irradiation, thereby inducing ICD in tumour cells. Compared to Mn^2+^ NPs, PB-Mn/OVA NEs treatment approximately doubled both the proportion of CD3^+^/CD8^+^ T cells and the number of memory T cells in mice, while demonstrating excellent long-term biosafety. More importantly, PB-Mn/OVA NEs significantly suppressed tumour growth and recurrence, whereas mice undergoing PTT alone (PB NPs group) exhibited progressive recurrence (77).

Another laser-based nanodelivery strategy for STING agonists leverages the photothermal effect to trigger phase transitions in thermoresponsive materials, enabling controlled drug release. For instance, Zhan et al. engineered a thermoresponsive liposome delivery platform modified with bromelain. Under NIR irradiation, FeS_2_ within the nanoplatform generates heat, causing the liposomes to melt and releasing cGAMP. In 4T1 mice, these nanoparticles degraded the tumour extracellular matrix through the synergistic action of bromelain and localised hyperthermia, thereby significantly enhancing the accumulation and deep penetration of STING agonists within tumour tissues. Compared to conventional LNPs, thermoresponsive liposomes not only effectively inhibited primary tumour growth but also significantly blocked the formation of distant metastatic lesions, whereas the LNPs group still exhibited noticeable metastatic foci (120). In another study, a thermoresponsive hydrogel nanoplatform (NvIH) significantly prolonged nanoparticle retention time at tumour sites by triggering a sol-gel phase transition, thereby enabling sustained activation of the STING pathway. A single injection of NvIH enabled STING agonists to persist within tumours for up to one week while effectively reducing acute systemic inflammatory responses caused by non-specific distribution. In a glioblastoma model, NvIH not only induced robust local anti-tumour immune responses but also stimulated systemic immune effects, significantly suppressing primary tumours, distant metastases, and tumour recurrence (125). Overall, light-responsive nanoplatforms achieve precise localized delivery and on-demand release of therapeutic agents to tumors through light-controlled mechanisms. They can synergistically induce ICD with PDT/PTT, thereby enhancing systemic antitumor immune responses. This approach partially addresses the limitations of endogenous responsive nanoplatforms, such as insufficient spatio-temporal controllability and non-specific activation. However, their therapeutic efficacy heavily relies on precise light source delivery and uniform irradiation. Constrained by light penetration depth in biological tissues, significant technical bottlenecks persist, particularly in treating deep-seated tumors. Future research urgently requires breakthroughs in enhancing light source tissue penetration, developing implantable or targeted optical devices, and optimizing the synergistic design of light-nanoplatform systems.

## 9 Radiation- and magnetic field- responsive nanoplatforms

Radiation-responsive nanocarriers have garnered significant attention for their ability to achieve precise drug release while synergising with radiotherapy. Recent studies have leveraged the radiosensitivity of ZIF-8 to construct an X-ray-responsive nanocarrier (Mn-ZIF-8) by doping Mn^2+^ and Mn^4+^, enabling synergistic combined application of radiotherapy and immunotherapy (135). Following transdermal administration, Mn-ZIF-8 acts as a radiosensitizer by inducing ICD and promoting the release of dsDNA. Concurrently, under the synergistic effects of the acidic TME and X-ray irradiation, Mn-ZIF-8 continuously releases Mn^2+^, which collaborates with dsDNA to activate the STING signalling pathway and induces systemic antitumour immunity. In a melanoma model, Mn-ZIF-8 combined with radiotherapy significantly enhanced the inhibitory effect of αPD-1 on both primary tumours and distant metastases compared to either X-ray alone or X-ray plus αPD-1 treatment (135). In another study, researchers crosslinked Mn-Au NPs with radiation-responsive diselenide-containing linkers to construct sMnAu NAs. These nanoparticles achieve efficient accumulation within tumour tissues while maintaining limited Mn^2+^ release during circulation, demonstrating excellent tumour targeting and biocompatibility. Following X-ray irradiation, they not only directly promote tumour cell death by generating ROS and inducing DNA damage, but also dissociate into Mn-Au nanoparticles, thereby activating the STING pathway and enhancing the anticancer effects of radiotherapy. In mouse models, the combination of sMnAu NAs with chemotherapy and radiotherapy significantly induced systemic immune responses, effectively suppressing tumour growth and distant metastasis (136). Finally, magnetic nanoparticles can be precisely guided to tumours under an external magnetic field, also providing a promising strategy for the targeted activation of the STING pathway. Huang et al. synthesised magnetic nanoparticles with Fe_34_ cores and Ca-doped MnCO_3_ shells for tumour antigen delivery. The nanoparticles can be actively and rapidly transported into the cytoplasm of DCs under an external magnetic field. In a mildly acidic environment, the nanoparticles degrade to release Ca^2+^, Mn^2+^, and tumour antigens, where Ca^2+^ regulates autophagy to enhance antigen cross-presentation, thereby promoting immune activation mediated by Mn^2+^ and tumour antigens (128). In summary, these studies demonstrate that both leveraging radiosensitisation effects and employing magnetic field-mediated active delivery strategies can significantly enhance the activation efficiency and immune cell infiltration levels of STING agonists at the tumour site. This, in turn, triggers a more potent systemic antitumour immune response.

## 10 Multi-responsive nanoplatforms

Although single-response STING-activated nanoplatforms have demonstrated favourable safety profiles and controlled drug release, multi-response nanocarriers can integrate multiple stimuli to achieve sequential or synergistic release. This further enhances the precision and controllability of drug delivery while effectively reducing biological toxicity (149). For example, light/ROS-responsive nanocarriers can trigger structural cleavage of the carrier through ROS generated by photosensitizers, thereby enabling temporally and spatially controlled drug release. Based on this approach, Xiao et al. co-encapsulated thioketone-linked CDN (TK-CDN) with Ce6 within TPP-modified liposomes, constructing the Lipo/TK-CDN/TPP/Ce6 nanoplatform. Under conditions without light stimulation, Lipo/TK-CDN/TPP/Ce6 remains stable within tumour cells, thereby reducing CDN-related toxic side effects and enhancing tumour targeting. Consequently, in melanoma models, both CDN alone and Lipo/TK-CDN/TPP/Ce6 demonstrated limited antitumour efficacy. However, under laser irradiation, this nanoplatform significantly inhibited tumour growth and more effectively activated the STING pathway. These findings indicate that the system enables on-demand release of CDN upon light stimulation, synergising PDT therapy with immunotherapy to induce potent systemic antitumour immune responses (96). Additionally, Tao et al. constructed a polymer composed of photodynamic monomers and thioketal-containing monomers for delivering the platinum-based drug 56MESS (NP ^PDT^-56MESS). Compared to the marked hepatotoxicity induced by free 56MESS, NP ^PDT-^56MESS-treated mice exhibited safety comparable to the untreated group, along with excellent tumour targeting and sustained tumour enrichment. In tumour-bearing mouse models, this nanoplatform induced DNA and mitochondrial damage through synergistic PDT and chemotherapy, thereby activating the STING pathway and demonstrating significant suppression of recurrent and metastatic melanoma (122).

In another study, researchers leveraged the GSH/pH-responsive properties of MnO_2_ with ester bonds to construct a TME-responsive nanoplatform. This system loaded the mitoxantrone–5-aminoacylpropionic acid prodrug (Mit-ALA), linked via an ester bond, onto iRGD-modified BSA-MnO_2_ nanoparticles (MBMA-RGD). In the 4T1 model, compared to free Mit-ALA and MBMA, MBMA-RGD predominantly accumulated in tumour tissues, with negligible distribution in other vital organs. Signal detection persisted at the tumour site 24 h post-injection, demonstrating excellent tumour targeting and retention capabilities alongside low systemic toxicity. Furthermore, under laser irradiation, MBMA-RGD significantly activated the STING pathway in the 4T1 model compared to other treatment groups, effectively enhancing CD4^+^ and CD8^+^ T cell infiltration in both primary and distant tumours (150). Moreover, Tian et al. developed a multi-responsive CRISPR nanoplatform using HA-modified hollow mesoporous manganese dioxide (H-MnO_2_) as the carrier. Within the TME, GSH and hyaluronidase trigger the release of CRISPR/Cas9 ribonucleoproteins (RNPs), the photosensitizer IR820, and MnO_2_. MnO_2_ decomposes H_2_O_2_ to generate O_2_ and Mn^2+^, thereby alleviating tumour hypoxia, activating the STING signalling pathway, and enhancing PDT-induced ROS generation. In addition, CRISPR/Cas9 RNPs target the *MTH1* gene to inhibit oxidative damage repair pathways, further amplifying ROS-mediated cytotoxicity. In liver cancer models, this nanosystem promoted ICD and DCs activation through STING pathway activation, while enhancing oxidative stress, ultimately eliciting a potent antitumour response (151). Overall, multi-responsive nanomedicines overcome the limitations of single-stimuli-responsive nanoplatforms by integrating multiple internal and external stimulus signals (**Table 4**). For instance, they address deficiencies such as insufficient tissue penetration under exogenous stimuli and limited adaptability to complex physiological environments under endogenous stimuli. This enables more precise and controllable drug delivery and release, effectively reducing systemic toxicity while significantly enhancing antitumour efficacy. However, compared to single-stimuli-responsive systems, multi-response nanoplatforms exhibit significantly increased complexity due to the integration of multiple functional components or multi-level structures. This further elevates the challenges in clinical translation processes, including manufacturing process standardization, quality control, and mass production.

**Table 4:** Representative multi-responsive nanoplatforms for STING activation.

Type	Synergistic therapy	Model	Nanoparticles	Content	Reference
GSH+ROS	SDT+CDT	Tumour‐bearing mouse	NP_MCA_	Ce6+Mno2+ADA	(152)
	Chemotherapy	Ovarian cancer	PTP@SR-717	Cisplatin+SR-717	(153)
GSH+pH	CDT+Chemotherapy	Lung/Breast cancer	MnO_2_@BSA@DOX	DOX+MnO_2_	(154)
	Radiotherapy	Colon/Breast cancer	MnAuNP-C&B	Mn+Au	(155)
	PD-1 inhibitor	Lung cancer	Mn-MSN@Met-M NPs	Mn+Metformin	(156)
pH+ROS	chemo/chemodynamic therapy	Mouse melanoma model	DPGM@anti-PD-L1	Gossypol+ anti-PD-L1+Mn	(97)
	PTT	Breast cancer	MnPB-MnO_x_ NPs	MnPB-MnO_x_	(157)
pH+Enzyme	—	Hepatocellular carcinoma	C-B-M-Mn nanovesicle platform	MSA-2+BAY-876+Mn	(158)
pH+Radiation	Radiotherapy	Breast cancer	Dy/Mn-P NPs	Dy^3+^+Mn^2+^	(159)
GSH+pH+ROS	Chemotherapy +PDT+PTT	Primary subcutaneous tumour	MBMA-RGD ISV	MIT+ALA+MnO_2_	(150)
	Gas therapy	Bilateral breast tumour	M‐RMH	Manganese carbonyl	(160)
GSH+pH+Light	Chemotherapy +PDT+PTT+CDT	Hepatocellular carcinoma	HMCuS/Pt/ICG@MnO_2_@9R-P201	ICG+Pt+ MnO_2_	(161)
	PDT	Breast tumour	DZ@A7	IR780+BD+Zn^2^	(162)
GSH+pH+Enzyme	PDT+PTT+CDT	Liver cancer	R820@H-MnO_2_-RNP@HA NPs	IR820+CRISPR/Cas9 RNPs	(151)
pH+ROS+Ultrasound	—	Mouse melanoma model	hMnO_2_@HA@NMA	NMA+Mn	(163)

SDT, sonodynamic therapy; CDT, chemodynamic therapy; PTT, Photothermal therapy; PDT, Photodynamic therapy; ADA, Adenosine deaminase; MIT, mitoxantrone; ALA, 5-aminolevulinic acid; BD, banoxantrone dihydrochloride; NMA, N-methyl-N-nitrosoaniline; CRISPR/Cas9 RNPs, CRISPR/Cas9 ribonucleoproteins.

## 11 Conclusion and perspectives

In summary, stimuli-responsive nanoplatforms enable controlled and exact drug release triggered by the TME or exogenous stimuli. This significantly enhances the delivery efficiency of STING agonists to tumour sites and the activation level of the STING pathway, while effectively reducing systemic toxicity caused by non-specific distribution. Furthermore, these nanoplatforms can integrate multiple therapeutic modalities, including chemotherapy, PDT/PTT, and immunotherapy, to synergistically amplify immune responses and oxidative stress effects. This approach further enhances STING signalling and remodels the immunosuppressive TME, offering a more promising therapeutic strategy for inhibiting tumour growth, metastasis, and postoperative recurrence. Nevertheless, their clinical translation still faces several critical challenges. First, most studies have been conducted in murine models, which cannot fully recapitulate the human immune system and tumour biology. Patient heterogeneity, along with the diversity of tumour types and microenvironmental characteristics, further limits the predictability of these nanocarriers in terms of targeting efficiency, therapeutic efficacy, and broad applicability. Furthermore, the current lack of unified and standardized *in vitro* and *in vivo* evaluation models not only limits systematic comparisons of different stimuli-responsive nanoplatforms in terms of drug loading capacity, release kinetics, and biodistribution, but also hinders prospective assessments of their long-term safety and immunogenicity. Finally, most nanocarrier studies stay at the laboratory stage, and challenges in large-scale manufacturing, stability, and compatibility with clinical-grade STING agonists present significant technical barriers, further constraining their feasibility for clinical translation. Looking ahead, there is an urgent need to establish standardized *in vitro* and *in vivo* evaluation systems. By systematically integrating standardized cell models, patient-derived three-dimensional tumor organoid systems, and reproducible small animal models, a representative tumour assessment framework can be constructed. This will enable systematic evaluation and comparative analysis of key parameters for various stimuli-responsive nanoplatforms within the same tumor type, including STING activation efficiency, drug release behavior, biodistribution, and safety. On this basis, the development of universal or modular STING-activating nanoplatforms adaptable to diverse tumour characteristics would provide a promising strategy for precise immune modulation and guide the optimised design of nanocarriers and combination therapies, including the selection and dosing of STING agonists, chemotherapeutic agents, radiotherapeutics, and photosensitizers. By integrating these strategies, it is expected to generate robust preclinical evidence, accelerate the clinical translation of STING-activating nanomedicines, and thereby provide new therapeutic and adjunctive options for resistant and hard-to-treat tumours. Taken together, stimuli-responsive STING-activating nanoplatforms represent a highly promising strategy for precise antitumour immune modulation. With continued interdisciplinary research, this field holds the potential to achieve groundbreaking clinical translation, ultimately reshaping the current landscape of cancer therapy.
